# Sweet proteins – Potential replacement for artificial low calorie sweeteners

**DOI:** 10.1186/1475-2891-4-5

**Published:** 2005-02-09

**Authors:** Ravi Kant

**Affiliations:** 1 Institute of Bioinformatics and Applied Biotechnology, ITPL, Bangalore-560066, India

**Keywords:** Sweet protein, Sweet taste receptor, Sweetener, T1R2-T1R3, Diabetes

## Abstract

Exponential growth in the number of patients suffering from diseases caused by the consumption of sugar has become a threat to mankind's health. Artificial low calorie sweeteners available in the market may have severe side effects. It takes time to figure out the long term side effects and by the time these are established, they are replaced by a new low calorie sweetener. Saccharine has been used for centuries to sweeten foods and beverages without calories or carbohydrate. It was also used on a large scale during the sugar shortage of the two world wars but was abandoned as soon as it was linked with development of bladder cancer. Naturally occurring sweet and taste modifying proteins are being seen as potential replacements for the currently available artificial low calorie sweeteners. Interaction aspects of sweet proteins and the human sweet taste receptor are being investigated.

## Sweet and taste modifying proteins

The prevalence of obesity and diabetes has increased dramatically in recent years in the United States, with similar patterns seen in several other countries including India [1] as well. Diabetes mellitus is a chronic disease caused by inherited or acquired deficiency in production of insulin by the pancreas or by the ineffectiveness of the insulin produced [2]. Artificial sweeteners like Saccharin, Aspartame, Cyclamate and AcesulfameK are used world-wide as low calorie sweeteners by patients affected by diseases linked to the consumption of sugar, e.g. diabetes, hyperlipemia, caries, obesity etc. but they have side effects such as psychological problems, mental disorders, bladder cancer, heart failure and brain tumors [3-7]. Sweet proteins have the potential to replace these artificial sweeteners, by acting as natural, good, low calorie sweeteners, as we know that proteins do not trigger a demand for insulin in these patients whereas sucrose does.

In humans, the sweet taste is mainly due to the recently discovered T1R2-T1R3 receptor [8-10], two of the three members of the T1R class [8-10] of taste-specific proteins hypothesized to function in combination as a heterodimer. The human T1R2-T1R3 receptor recognizes natural and synthetic sweetness and T1R1-T1R3 recognizes umami taste [11,12]. So far there are seven known sweet and taste-modifying proteins, namely Brazzein [13], Thaumatin [14], Monelin [15], Curculin [16], Mabinlin [17], Miraculin [18] and Pentadin [19]. Properties and characteristics of these proteins are illustrated in Table 1. The key residues on the protein surface responsible for biological activity have not yet been identified with certainty for any of these proteins [20]. Monellin was found to be 100000 times sweeter than sucrose on a molar basis [21], followed by Brazzein and Thaumatin which are 500 times [13] and 3000 times sweeter then sucrose [14] respectively (the latter two on a weight basis). All of these proteins have been isolated from plants that grow in tropical rainforests. Although most of them share no sequence homology or structural similarity, Thaumatin shares extensive homology with certain non-sweet proteins found in other plants [15].

The potential industrial applications of these proteins are the low calorie sweetener industry and the cola, snacks, food and chocolate industries.

## Brazzein

Brazzein is the smallest, most heat-stable [13] and pH-stable member of the set of proteins known to have intrinsic sweetness. The protein, consisting of 54 amino acid residues, is reported to be between 500 and 2000 times sweeter than sucrose [22] and represents an excellent alternative to available low calorie sweeteners. It was originally isolated from the fruit of an African plant *Pentadiplandra brazzeana *Baillon [23]. Heat and pH stability of the protein make it an ideal system for investigating the chemical and structural requirements of a sweet-tasting protein. Based on the wild-type brazzein, 25 mutants were produced to identify critical regions important for sweetness. To assess their sweetness, psychophysical experiments were carried out with 14 human subjects. First, the results suggest that residues 29–33 and 39–43, plus residue 36 between these stretches, as well as the C-terminus are involved in the sweetness [24]. Second, charge plays an important role in its interaction with the sweet taste receptor [24].

## Thaumatin

The thaumatins are a class of intensely sweet proteins isolated from the fruit of the tropical plant *Thaumatococcus danielli*. The protein crystallizes in a hexagonal lattice after a temperature shift from 293 to 277 K. The structure has been solved at 1.6 Å resolution. Its fold was found to be identical to that found in three other crystal forms grown in the presence of crystallizing agents of differing chemical natures [25]. It consists of 207 amino acid residues with eight intramolecular disulfide bonds and contains no free cysteine residues. It aggregates upon heating at pH 7.0 above 70 degrees C, whereupon its sweetness disappears [26,27]. The protein is approximately 10000 times sweeter than sugar on a molar basis [28]. It is a protein that tastes intensely sweet only to Old World monkeys and to higher primates, including man [29], as it has been found that the protein binds to certain elements in taste pores of Rhesus monkey foliate papillae [30]. Thaumatin has been approved for use in many countries as both a flavor enhancer and a high-intensity sweetener [31].

## Monellin

Monellin, a sweet protein, consists of two noncovalently associated polypeptide chains, an A chain of 44 amino acid residues and a B chain of 50 amino acid residues [32]. The protein can be purified from the fruit of *Dioscoreophyllum cumminsii *grown in West Africa and is approximately 100,000 times sweeter than sugar on a molar basis and several thousand times sweeter on a weight basis [28]. Single-chain monellin (SCM), which is an engineered 94-residue polypeptide, has proven to be as sweet as native two-chain monellin, and is more stable than the native monellin at high temperature and in acidic environments [33]. Native monellin is relatively sensitive to heat or acid treatment, which may cause separation of the sub-units and denaturation of the protein. Despite misgivings about the stability of the protein to heat and acid, downstream processes have been established. Its D-enantiomer has been crystallized and analyzed by X-ray crystallography at 1.8 Å resolution. Two crystal forms (I and II) were found under crystallization conditions similar, but not identical, to the crystallization conditions of natural L-monellin [34]. One NMR study of a non-sweet analog in which the Asp^B7 ^of protein was replaced by Abu^B7 ^(L-2-Aminobutylicacid), showed similar 3-dimensional structures of these two proteins, indicating that the lack of the beta-carboxyl group in the Abu^B7 ^analog is responsible for the loss of sweetness [35]. Recent research on identifying binding sites on the receptor by means of structure-taste relationships, found that four monellin analogues, [AsnA16]-, [AsnA22]-, [GlnA25]-, and [AsnA26]-monellin were 7500, 750, 2500, and 5500 times as sweet as sucrose on a weight basis, respectively. Thus, among them, [AsnA22]-monellin and [GlnA25]-monellin were less sweet than the native monellin [36].

## Curculin

Curculin which is extracted from *Curculigo latifolia *acts as a good low calorie sweetener. Its maximum sweetness is equal to 0.35 M of sucrose. It has taste modifying abilities since water and sour substances elicit a sweet taste after consumption of curculin [37]. There is no other protein currently available with both sweet taste and taste modifying abilities [38]. The taste modifying activity of the protein (discussed below) remains unchanged when it is incubated at 50°C for 1 hr between pH 3 and 11 [39].

The molecular weight of Curculin was determined by low angle laser light scattering and was found to be 27800 [38]. Its three-dimensional model has been built from the X-ray coordinates of GNA, a mannose-binding lectin from snowdrop (*Galanthus nivalis*) [38]. The three mannose-binding sites present in GNA were found in curculin but were not functional. Some well exposed regions on the surface of the three-dimensional model of the said protein could act as epitopes responsible for the sweet-tasting properties of the protein [40]. The protein can be crystallized by the vapor diffusion method using polyethylene glycol 400 as a precipitant. The crystals belong to orthorhombic space group P2(1)2(1)2(1) with unit cell dimensions: a = 105 Å, b = 271 Å, c = 48.7 Å. The crystals diffract X-rays to resolution of 3.0 Å and are suitable for X-ray crystallographic studies [41].

## Mabinlin

Mabinlin is a sweet protein with the highest known thermostablility [42]. It is derived from *Capparis masaikai *and its sweetness was estimated to be around 400 times that of sucrose on weight basis. It consists of an A chain with 33 amino acid residues and a B chain composed of 72 residues. The B chain contains two intramolecular disulfide bonds and is connected to the A chain through two intermolecular disulfide bridges [43]. Its heat stability is due to the presence of these four disulfide bridges [44]. The sweetness of Mabinlin-2 is unchanged after 48 hr incubation at boiling point [17]and of Mabinlin-3 and -4 are unchanged after 1 hr at 80°C [45].

## Miraculin

Miraculin is a taste-modifying protein that belongs to the class of sweet proteins. It is extracted from *Richadella dulcifica *an evergreen shrub native of West Africa. The protein is a single polypeptide with 191 amino acid residues [46]. It modifies the sweet receptor in such a way that it can be stimulated by acid [47]. Thus, miraculin has the unusual property of modifying sour taste into sweet taste [46].

Taste-modifying protein modifies the sweet taste receptor on binding and this behavior of these proteins is responsible for modification in taste of sour substance [46,47]. All acids (which are normally sour) taste sweet after consumption of these proteins. The effects of these proteins exist for around half an hour after consumption and intake of any sour substance will therefore taste sweet during this period of time. The taste buds come to there normal state with time.

## Pentadin

Pentadin is a sweet protein extracted from the plant *Pentadiplandra brazzeana*, a shrub found in tropical forests of a few African countries. Not much information is available about the protein despite its isolation several years ago, in 1989 [48]. The protein was reported to be around 500 times sweeter then sucrose on a weight basis. It also consists of subunits coupled by disulfide bonds [49].

## Interaction of sweet proteins with their receptor

Humans detect taste with taste receptor cells. These are clustered in taste buds. Each taste bud has a pore that opens out to the surface of the tongue enabling molecules and ions taken into the mouth to reach the receptor cells inside. There are five primary taste sensations salty, sour, sweet, bitter and umami. Sweet and umami (the taste of monosodium glutamate) are the main pleasant tastes in humans. T1Rs are mammalian taste receptors that assemble two heteromeric G-protein-coupled receptor complexes T1R1+T1R3, an umami sensor, and T1R2+T1R3, a sweet receptor [50].

Sweet and taste-modifying proteins interact with the T1R2-T1R3 receptor with a different mechanism compared to small molecular weight compounds [51]. Recently, it has been shown that the T1R2-T1R3 receptor has many characteristics similar to the mGluR [52], apart from some minor differences in the active site region.

**Figure 1:**
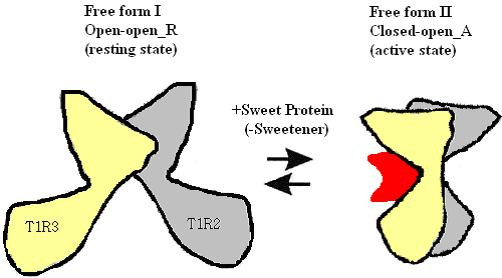
Diagrammatic representation of the T1R2-T1R3 receptor showing possible stabilization by binding of a sweet protein to a secondary binding site on the surface of free form II. The sweet protein is represented in red on the left part of free form II, preventing it reverting to free form I. (adapted from references 52 and 54)

The major work by Kunishima et al. [52] solving the crystal structure of the N-terminal active site region of the subtype 1 of mGluR both free and complexed with glutamate has helped a lot in understanding the mechanism of interaction between ligand and T1R2-T1R3 receptor. Their structural work on mGluR and its N-terminal domain [52,53] showing considerable conformational change induced by the glutamate complexation. The 'Active' and 'resting' conformations of m1-LBR, an extracellular ligand binding region of mGluR, is modulated by the dimer interface. The protomer can form 'open' or 'closed' confirmations and is made up of two domains namely LB1 and LB2. The population of active conformers depends on ligand binding, i.e. the so called 'closed-open_A'. The ligand-free receptor exists as two different structures, free form I (open-open_R), the 'resting' conformation with two open protomers and free form II (closed-open_A), nearly identical to the complexed form (Figure 1, references 52, 54). The mechanism suggested by these structures is that the receptor is in dynamic equilibrium, and that ligand binding stabilizes the 'active' dimer. There are thus two ways, in principle, to activate the receptor: first, to complexate form I with the proper ligand (glutamate for the mGluR, aspartame or any other small molecular weight sweetener for the T1R2-T1R3 receptor) and second, by shift the equilibrium between free form I and free form II in favor of free form II.

The exact mechanism of interaction of sweet proteins with the T1R2-T1R3 sweet taste receptor has not yet been elucidated [51]. Low molecular mass sweeteners and sweet proteins interact with the same receptor, the human T1R2-T1R3 receptor[52]. Studies have shown that the T1R3 receptor protein is encoded by the Tas1r3 gene involved in transduction of sweet taste [55].

Recently it has been found that T1R3-independent sweet- and umami-responsive receptors and/or pathways also exist in taste cells [56].

## Conclusion and scope of further work

As it has been found that sweet proteins are thousands of times sweeter than sucrose and are of low calorie value, these proteins can be used as natural low calorie sweeteners by people suffering from diseases linked to consumption of sugar e.g. obesity, diabetes and hyperlipemia.

Candidate proteins can be checked for biological activity with the human taste receptor. Also mutations can be induced in candidate sweet proteins to analyze changes in their physical, chemical and biological properties. The work can be taken forward by solving the structures of the proteins and taste receptors with a view to increasing the efficiency of these sweeteners.

## Figures and Tables

**Table 1:** Comparison of thaumatin, monellin, mabinlin, pentadin, brazzein, curculin and miraculin.

	**Thaumatin**	**Monellin**	**Mabinlin**	**Pentadin**	**Brazzein**	**Curculin**	**Miraculin**
Source	Thaumatococcus danielli Benth	Dioscoreophyllum cumminsii Diels	Capparis masakai Levl	Pentadiplandra brazzeana Baillon	Pentadiplandra brazzeana Baillon	Curculingo latifolia	Richadella dulcifica
Geographic distribution	West Africa	West Africa	China	West Africa	West Africa	Malaysia	West Africa
Variants	I, II, a, b, c^a^	-	I, II- a, III, IV^a^	-	-	-	-
Sweetness factor (weight basis)	3000	3000	100	500	2000	550	-
Molecular mass (active form, kDa)	22.2	10.7	12.4	12.0^b^	6.5	24.9	98.4
Amino acids	207	45 (A chain) 50 (B chain)	33 (A chain) 72 (B chain)	?	54	114	191
Active form	Monomer	Dimer (A + B)	Dimer (A + B)	?	Monomer	Dimer (A + A)	Tetramer (A+A+A+A)

Source: Adapted from Kurihara (1994). ^a^At least five different forms of thaumatin (Lee et al., 1988) and four different forms of mabinlin (Nirasawa et al., 1994) have been identified. ^b^A chromatographic fraction containing a 12-kDa protein was sweet. This same fraction, when subjected to electrophoresis under non-reducing conditions showed bands in the region between 22 and 41 kDa, suggesting the presence of subunits.
